# The Theories of the Development of Students: A Factor to Shape Teacher Empathy From the Perspective of Motivation

**DOI:** 10.3389/fpsyg.2021.736656

**Published:** 2021-11-16

**Authors:** Yabo Ge, Weijian Li, Fangyan Chen, Sumaira Kayani, Guihua Qin

**Affiliations:** ^1^Department of Psychology, Zhejiang Normal University, Jinhua, China; ^2^Key Laboratory of Intelligent Education Technology and Application of Zhejiang Province, Zhejiang Normal University, Jinhua, China; ^3^Teachers College, Jinhua Polytechnic, Jinhua, China

**Keywords:** teacher empathy, empathic propensity, motivation, empathic motivation, teacher education

## Abstract

Empathy represents an essential prerequisite for developing effective interpersonal behavior and maintaining interpersonal relationships. Education is a result of teacher-student interaction, and, therefore, it is worth noting that teaching empathy is critical for the development of students and the professional growth of teachers. Recently, researchers began to explore the influential factors of empathy (e.g., empathic mindsets) based on motivation. Beyond their empathic attitudes, teachers also have a mindset toward the development of students. A survey study was adopted to explore the relationship between the theories of the growth of students and teacher empathy. Four hundred and eighty-four Chinese teachers completed the student development scale, the teacher empathic motivation scale, and the teacher empathy scale. The mediation model results showed that the theories of the development of students could significantly predict teacher empathy and teacher empathic motivation. The teacher empathic motivation mediated the positive relationship between the theories of the development of students and teacher empathy. This study proposes a new concept and method for teacher empathy intervention in future.

## Introduction

Empathy, an interpersonal phenomenon, refers to sharing in and understanding the thoughts, and feelings of other people, and caring for their welfare (Zaki, 2014; Preston and Waal, 2017; Yang et al., 2018; Weisz and Cikara, 2021). It is broadly believed that empathy is a critical ingredient in interpersonal processes (Main et al., 2017; Amicucci et al., 2021). Moreover, related studies have noted an impairment of social functioning consequent upon the empathy deficit in a series of neuropsychiatric conditions (Shimoni et al., 2012; Laisney et al., 2013; Schreiter et al., 2013; Yang et al., 2018). As is generally known, teaching is a social interaction that involves a pupil and a teacher (Holper et al., 2013), which is also inseparable from empathy (Swan and Riley, 2015). Teacher empathy, which involves cognitive and affective elements (Tettegah and Anderson, 2007; Swan and Riley, 2015; Goroshit and Hen, 2016), involves comprehensively understanding the situation of students sharing the positive and negative emotions of students, and expressing care for the students through actions (Berkovich and Eyal, 2015; Meyers et al., 2019; Ronen, 2020). Currently, there is an enhanced awareness of the importance of teacher empathy in teacher education (Swan and Riley, 2015).

### The Value of Culturing for Teacher Empathy

It is generally accepted that teacher empathy is significantly correlated with the development of students and teachers. Accumulating evidence suggests that, on the one hand, teacher empathy can promote the academic achievement of students (Cadima et al., 2010; Warren, 2018; Ronen, 2020), their motivation for learning (Cooper, 2004), teacher-student relationship (Wubbels and Brekelmans, 2005; Stojiljković et al., 2012), and the overall classroom atmosphere (Cooper, 2010). Additionally, teacher empathy is not merely meant to encourage student engagement in learning but to help achieve social justice across diverse backgrounds (Bullough, 2019). Therefore, arguably, education is not complete without teacher empathy; if not, teachers are teaching to transmit content instead of teaching the students (Swan and Riley, 2015). On the other hand, teacher empathy plays a vital role in promoting the development of students and is a crucial feature of the identity of teachers, which can promote their professional growth (Stojiljković et al., 2012; Zhu et al., 2019). For example, empathy effectively facilitates the specialization of teachers through establishing positive teacher-student relationships and a relaxed teaching atmosphere (Stojiljković et al., 2012). Moreover, some investigators noted that empathy had been long considered central to the teaching profession (Jaber et al., 2018).

Overall, empathy-building interventions for teachers have a clear adaptive function for social interaction, both for the students and the teachers. Thus far, empathy interventions have focused on building perspective-taking strategies and increasing empathy-expression strategies (Weisz and Zaki, 2017). However, these intervention techniques have not yielded impressive results (Waller et al., 2020). Therefore, it is essential to identify other factors that shape teacher empathy.

### Empathy and Motivation

Empathy is not always automatic but is rather context-dependent (Zaki, 2014). Furthermore, empathy, like many other psychological phenomena, involves a motivational component (Weisz et al., 2020). Empathic motivation is goal-directed, an internal force that drives people toward and away from social connections (Weisz and Zaki, 2018). More importantly, Keysers and Gazzola (2014) proposed that the ability-propensity distinction is crucial to characterizing empathy. According to this theory, there may be variations in empathy owing to not only the ability difference but also the motivation difference. Similarly, Ferguson et al. (2020) suggested that empathy is a choice and can be evoked in multiple ways. Therefore, similar to empathic ability, empathic motivation serves a significant role in the process of empathy. The relevant theory of empathic motivation provides a new insight into empathy culturing as to date, many empathic cultivations focus on developing the ability of people to empathize via experience-based and expression-based interventions (Weisz and Zaki, 2017).

### Teacher Empathy: The Potential Role of Teachers’ Beliefs

It is novel to identify the factors that shape teacher empathy based on motivation. Belief is one of the most critical factors that influence motivation, such as the achievement goal theory (Dweck, 1996). Recently, more and more researchers have focused more on beliefs, which play an essential role in empathy and empathic motivation, and have gained many advances (Schumann et al., 2014; Weisz et al., 2020; Gandhi et al., 2021). For example, Weisz et al. (2020) found that the participants who had a more robust belief about the malleability of empathy exhibited greater empathic motivation and empathic accuracy. Similarly, Gandhi et al. (2021) reported that individuals who believed that empathy was changeable exhibited more empathetic behavior (less aggression).

Likewise, various educational researchers suggest that teachers’ beliefs affect their classroom practice (Kagan, 1992; Fang, 1996; Mansour, 2009). For example, Wang and Yang (2021) found that most pre-service STEM teachers hold the reality of development and the possibility of developing beliefs about migrant students. Moreover, teachers’ beliefs can also affect student motivation through classroom practice. Heyder et al. (2020) suggested that the more teachers believed that math requires innate ability, the lower the intrinsic motivation of students with low-achieving was. These studies also show that there is an intimate relationship between teacher beliefs and teacher practices. Therefore, just like in the general domain of empathy studies, we believe that the empathy and empathic motivation of teachers, belonging to the practices of teachers, may also be influenced by the beliefs of the teachers. As mentioned above, empathic motivation is a crucial influencing factor of empathy. Collectively, we speculate empathic motivation would play a mediating role in the relationship between the beliefs and empathy of teachers.

### The Current Study

As stated above, many investigators have paid increasing attention to the role of beliefs in empathy and empathic motivation in recent years (Schumann et al., 2014; Weisz et al., 2020). For example, Schumann et al. (2014) defined the empathic mindset as a malleable mindset through which believing empathy can be developed and a fixed theory through which believing empathy cannot be set. The results suggest that people who had a malleable mindset expended greater empathic motivation in challenging contexts than those who believed in a fixed theory. Similarly, as alluded to earlier, Weisz et al. (2020) found the same results. These findings suggested that empathic belief, a motivation-based intervention, is an essential factor that shapes empathy.

Fives and Buehl (2012) divided the beliefs of teachers into six categories, in which beliefs about students refer to the views about linguistic difference, capacity, learning, and the development of students. Researchers believe that beliefs about students are the most critical influencing factor of teacher practices, which is in a relatively central position (Wang and Yang, 2021). In other words, teachers, in the educational context, have not only their beliefs about themselves but also beliefs about students (e.g., the development of student abilities). According to Dweck et al. (1995) the theory of mindsets, teachers may have two different beliefs about the development of the ability of students. Teachers with malleable beliefs of the ability of students may think that the ability of students is unstable and can be enhanced through acquired efforts. On the contrary, teachers with fixed mindsets of the abilities of students may believe that their abilities are fixed and unchangeable. This raises the question of whether teacher empathy and teachers’ empathic motivation are influenced by their beliefs about the development of students.

**FIGURE 1:**
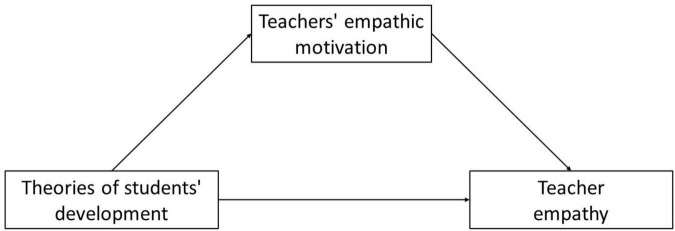
The conceptual model of the relationships between teachers’ beliefs about Students’ development, teachers’ empathic motivation, and teacher empathy.

Similar to beliefs about oneself, in this study, we expected teacher beliefs about the development of students to play an essential role in the empathic motivation and empathy of teachers. A mediation model was established to test these hypotheses; Figure 1 presents the conceptual model. This study’s primary contribution was to first investigate the role of teacher beliefs about the ability development of students under teacher empathy based on the motivation perspective.

## Materials and Methods

### Participants

Based on the model complexity (e.g., *n* = 5–10 per estimated parameter) (Bentler and Chou, 1987), the reasonable sample size for this investigation was between 285 and 570. A total of 495 Chinese teachers were recruited from kindergarten, primary school, junior high school, and senior high school in Zhejiang province. Eleven participants were excluded from the data screening process as they chose the same option on all the scales. The valid sample included 484 Chinese teachers [84.1% female; 67 no-titles (13.8%), 163 secondary title (33.7%), 194 primary title (40.1%), and 67 senior title (13.8%); 18 kindergarten teachers (3.7%), 320 primary school teachers (66.1%), 83 junior high school teachers (17.1%), and 63 senior high school teachers (13.0%)]. The average age was (mean ± SD) 35.81 ± 8.27 years old; the average teaching experience was (mean ± SD) 14.78 ± 10.98 years.

### Measures

#### Demographic Information Questionnaire

The sociodemographic characteristics of the participants were evaluated using a questionnaire that included the following information: gender, age, teaching experience, and title (i.e., “What is your teaching experience in terms off years?”).

#### Theories of the Development of Students

To construct a more accurate evaluation method of the teacher’s mindset regarding the development of students (TOS), we adapted a three-item measure from an existing measure of the implicit theory of personality (Dweck et al., 1995) and theories of empathy (Schumann et al., 2014). The questionnaire was scored on a 7-point scale (1 = strongly disagree, 7 = strongly agree), followed by three statements related to the teacher’s mindset of the ability of students. The statements were: “The level of Students’ learning ability is stable to some extent, and students are not able to change it. Students can indeed learn new knowledge, but they cannot improve their learning ability. That is Students’ learning ability is unlikely to change.” After statistical analysis, the internal consistency of the questionnaire was highly reliable (Cronbach’s α = 0.86).

#### Motivation for Teacher Empathy

Empathic motivation is a very abstract concept with motivational content or driving direction. This study focuses on the driving direction of motivation. It is self-edited to be suitable for the educational situation in which teachers are approaching the intensity of the empathic motivation, that is, the teachers’ empathic motivation (TEM) questionnaire. The questionnaire is adapted from the study of Schumann et al. (2014), and includes three statements (e.g., “when a student is in a bad mood,” “I want to know what they are thinking that moment,” “I am willing to share in their bad feelings,” and “I want to comfort them.”). Participants responded to the questionnaire on a 7-point agreement scale (1 = strongly disagree, 7 = strongly agree). A statistical analysis showed that this questionnaire has a high internal consistency coefficient (Cronbach’s α = 0.83).

#### Teacher Empathy

The Chinese version of the interpersonal reactivity index (IRI-C) (Huang et al., 2011) was revised and normalized for Chinese (Sun et al., 2017) to assess empathy (Likert 5-point, from 0 to 4). This scale version is widely used in Chinese culture (Sun et al., 2017; Chen et al., 2018). To construct a more accurate evaluation method, we composed an empathy questionnaire (IRI for Chinese teachers’ empathy, IRI-CT) to evaluate teacher empathy (TE) based on IRI-C. Specifically, we changed the statements of IRI-C to fit the current study purpose (i.e., empathic target). For example, we revised IRI-C “I often have tender, concerned feelings for people less fortunate than me.” and “I sometimes find it difficult to see things” from the “other person’s point of view.” to “I often have tender, concerned feelings for students less fortunate than me.” and “I sometimes find it difficult to see things from the ‘Students” point of view” (see Supplementary Appendix).

IRI-CT, which is similar to the IRI-C, also assesses four aspects of teacher empathy, namely, empathic concern (i.e., TE-EC, seven items), perspective-taking (i.e., TE-PT, seven items), fantasy (i.e., TE-FS, seven items), and personal distress (i.e., TE-PD, seven items). Participants rated their agreement or disagreement with 28 items on a 7-point scale (1 = does not describe me well, 7 = describes me very well). To validate the four-factor model, a confirmatory factor analyses was used to assess the model fit (*x*^2^/*df* = 3.98, RMSEA = 0.07, CFI = 0.73, TLI = 0.70, SRMR = 0.10), which shows a moderate structure validity. The correlations between the IRI-CT subscales and related constructs were significant (Zhao et al., 2018). For instance, TE-EC was negatively correlated with verbal aggression (the subscale of AQ) (*r* = −0.15, *p* < 0.001), difficulty in identifying one’s own feelings (the subscale of TAS-20) (*r* = −0.14, *p* < 0.01) and positively correlated with prosocial tendencies (PTM) (*r* = 0.41, *p* < 0.001), relational needs (the subscale of BPNS) (*r* = 0.30, *p* < 0.001). An internal consistency analysis revealed that the adapted scale of IRI has moderate reliability (α = 0.74). All the subscales of the IRI-CT demonstrated good internal consistencies (ranging from 0.57 to 0.71, see Table 1), which was consistent with other studies based on the Chinese versions of IRI (Zhao et al., 2021).

**TABLE 1:** Descriptive statistics and partial correlations for key variables.

Variable	*M* ± *SD*	1	2	3	4	5	6	7
1 Theories of the development of students	12.50 ± 3.36	**0.86**						
2 The empathic motivation of teachers	16.87 ± 2.99	0.31***	**0.83**					
3 Teacher empathy (TE)	134.88 ± 13.64	0.13***	0.43***	**0.74**				
4 Teacher empathy-EC (TE-EC)	38.38 ± 4.84	0.25***	0.51***	0.64***	**0.57**			
5 Teacher empathy-PT (TE-PT)	37.88 ± 4.95	0.37***	0.55***	0.51***	0.54***	**0.70**		
6 Teacher empathy-FS (TE-FS)	28.49 ± 6.60	0.03	0.18***	0.74***	0.20***	0.07	**0.71**	
7 Teacher empathy-PD (TE-PD)	30.13 ± 5.89	−0.24***	–0.08	0.53***	–0.02	−0.20***	0.37***	**0.71**

Cronbach’s alpha for each scale is in bold and in the diagonals. ***p < 0.001.

### Procedures

This study was conducted through a web-based survey via a Chinese survey website.^1^ All the participants in the questionnaire survey were volunteers and were asked to read the introduction to the study. The participants were then immediately instructed to fill out a demographic information questionnaire, and complete the whole task carefully. Data including demographic information, theories of the development of students, the empathetic motivation of teachers, and the subscales, such as TE-EC, TE-PT, TE-FS, and TE-PD were collected. The survey could not be submitted if any questions had not been answered, like other studies (Zhao et al., 2021). Hence, there were no missing values. They took approximately 8 min to complete all the assessments. Once they completed the questionnaire, the participants were debriefed about the purpose of this study and thanked for their participation. The Zhejiang Normal University Review Board approved the current research procedures.

### Data Analysis

The data analysis was as follows. First, SPSS (version 23.0) was used to calculate the descriptive statistics and partial correlations of crucial variables in this study. One recent cross-cultural study reported the culture-sex interaction effect for both trait and state empathy with Australian and Chinese subjects (Zhao et al., 2021). Therefore, partial correlation analyses (i.e., to control for the following covariates: gender, age, teaching experience, and title) were conducted to examine the relationships between the theories of the development of students (TOS), the empathic motivation of teachers, teacher empathy (TE), the empathic concern of teacher empathy (TE-EC), the perspective-taking of teacher empathy (TE-PT), the fantasy of teacher empathy (TE-FS), and the personal distress of teacher empathy (TE-EC). Second, the hypothesized mediation model was tested using the PROCESS macro for SPSS (Hayes, 2013). The hypothesized mediation [the theories of the development of students → the empathic motivation of teachers (mediator) → teacher empathy, TE-EC, TE-PT, TE-FS, and TE-PD, respectively] was tested using model 4 of the PROCESS macro for SPSS (version 3.3, see Hayes, 2013). For the current analyses, a meaningful indirect effect was identified depending on whether zero was outside the 95% confidence interval (CI) of the indirect effect (Field, 2013). Gender, age, teaching experience, and title were controlled for. Further, the models were tested using 5,000 bootstrap samples.

## Results

### Preliminary Analyses

Table 1 shows the descriptive statistics and the partial correlation analyses (i.e., to control for covariates) for the key variables. As expected, the theories of the Students’ development were positively correlated with the empathic motivation of teachers, teacher empathy, TE-EC and TE-PT, but negatively correlated with TE-PD. Additionally, the empathic motivation of teachers was significantly and positively related to teacher empathy, TE-EC, TE-PT, and TE-FS. The above preliminary analyses indicate a close relationship with the theories of Students’ development, empathic motivation, and teacher empathy (including subscales), which is also the basis of the mediation model analysis.

### Mediation Model

**FIGURE 2:**
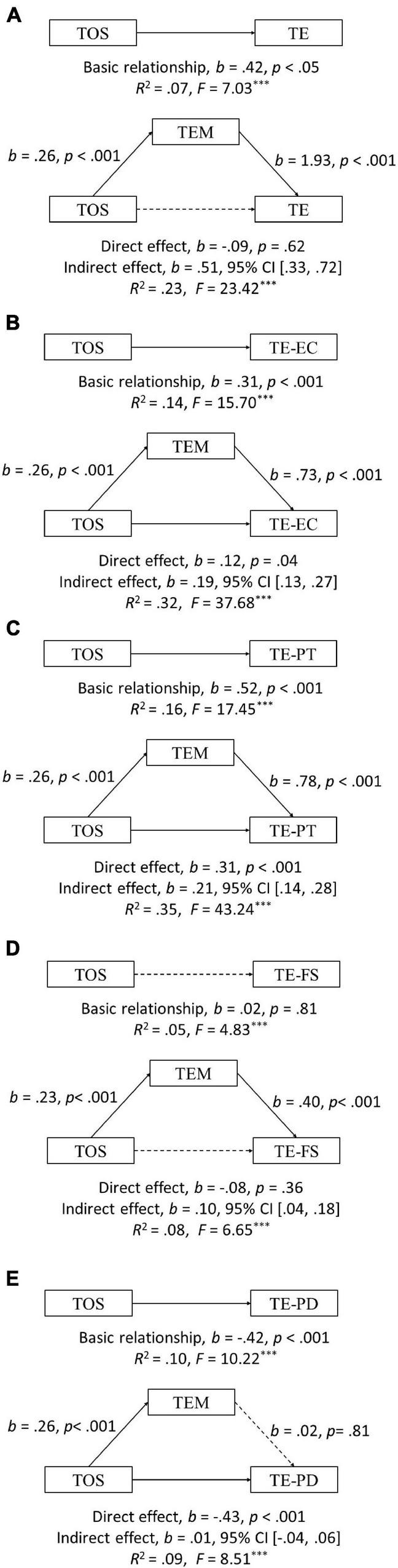
Models of teacher empathic motivation (TEM) as a mediator variable for the relationship between the theories of student development (TOS), teacher empathy (TE; **A**), empathic concern of teacher empathy (TE-EC; **B**), perspective taking of teacher empathy (TE-PT; **C**), fantasy of teacher empathy (TE-FS; **D**) and personal distress of teacher empathy (TE-PD, **E**). Non-standardized coefficients are reported. The solid lines represent significant coefficients, and the dashed line means insignificant effects. ***p < 0.001.

Two univariate outliers (i.e., an outlier for each of TEM, TE-PD) were identified (z-scores > 3.29) and excluded (Zhao et al., 2019, 2020). As indicated in Figure 2A, for teacher empathy, the total effects model with the theories of Students’ development and beforementioned covariates (i.e., gender, age, teaching experience, and title) showed that the regression coefficient for the theories of the development of the students was significant (*b* = 0.42, *SE* = 0.18, *p* = 0.02, 95% *CI* = [0.06,0.78]), which indicated that the total effect of the theories of the development of the students on teacher empathy was significant. Further, the mediation model controlled for the covariates was tested. The results showed that the empathic motivation of teachers was significantly predicted by the theories of the development of the students (*b* = 0.26, *SE* = 0.04, *p* < 0.001, 95% *CI* = [0.19,0.34]) and that teacher empathy was significantly predicted by the empathic motivation of teachers (*b* = 1.93, *SE* = 0.19, *p* < 0.001, 95% *CI* = [1.55, 2.31]). Further, the predictive relationship between the theories of Students’ development and teacher empathy was not significant when teachers’ empathic motivation was included in the regression equation (*b* = −0.09, *SE* = 0.17, *p* = 0.62, 95% *CI* = [−0.43,0.26]). The indirect effect of the empathic motivation of teachers was (*b* = 0.51, *SE* = 0.10, 95% *CI* = [0.33,0.72]). There is a meaningful indirect effect, as mentioned above when the zero was outside the 95% confidence interval (CI) of the indirect effect (Field, 2013). Therefore, the relationship between the theories of Students’ development and teacher empathy was mediated by the empathic motivation of teachers.

The abovementioned analytic approach will also be used for subscales of teacher empathy including TE-EC, TE-PT, TE-FS, and TE-PD. The results showed that the empathic motivation of teachers has a mediating effect between the theories of Students’ development and teacher empathy subscales except for TE-PD; namely, TE-EC (the indirect effect *b* = 0.19, *SE* = 0.04, 95% *CI* = [0.13,0.27], see Figure 2B), TE-PT (the indirect effect *b* = 0.21, *SE* = 0.04, 95% *CI* = [0.14,0.28], see Figure 2C), TE-FS (the indirect effect *b* = 0.10, *SE* = 0.03, 95% *CI* = [0.04,0.18], see Figure 2D) and TE-PD (the indirect effect *b* = 0.01, *SE* = 0.02, 95% *CI* = [−0.04,0.06], see Figure 2E).

## Discussion

Teacher empathy plays a vital role in the Students’ development and the professional development of teachers (Stojiljković et al., 2012). It is the foundation of the empathy culture to identify the factors that shape this psychological variable. This study investigates the role of teachers’ beliefs about Students’ development in the empathic motivation and empathy of teachers via the mediation model. Our finding suggests that the theories of Students’ development were positively associated with the empathic motivation and teacher empathy. The empathic motivation of teachers was positively associated with teacher empathy, and mediated the positive relationship between the theories of Students’ development and the teacher empathy. Therefore, the belief of teachers about Students’ development may be an essential characteristic of empathic motivation that contributes to teacher empathy in the educational context.

### The Theories of the Development of Students and Teacher Empathy

To the best of our knowledge, no previous study investigates the direct link between the theories of Students’ development and teacher empathy. However, some evidence from previous research indicates that the beliefs of teachers affect teacher practice, such as teaching effectiveness (Jordan et al., 2010). Additionally, Lavigne (2014) suggests that teachers develop beliefs about students as part of the teacher identity process, and focus more on student understanding and achievement. Besides, the research found that the teachers’ self-confidence was significantly associated with their Students’ self-confidence (Larina and Markina, 2020). Consistent with these previous findings among educational context, we observed a significant association between the teachers’ belief (i.e., the theories of Students’ development) and teachers’ practices (i.e., teacher empathy and three subscales). In other words, teachers who have a malleable mindset of the ability of students were more likely to exhibit high empathic concern and perspective-taking, and low personal distress toward students, while fantasy does not. One possible explanation for this is that fantasy was designed to evaluate a person’s propensity to appreciate the emotions of fictitious characters in movies, plays, or books (i.e., “When I watch a good movie, I can very easily put myself in the place of a leading character”) (Davis, 1980); some researchers assert that fantasy does not evaluate empathy *per se* (Baron-Cohen and Wheelwright, 2004) and hence exclude it from the data analysis (Zhao et al., 2019). Therefore, fantasy may not accurately reflect teacher empathy, which is to comprehensively understand the situation of students, share the positive and negative emotions of students, and express care for them through their actions (Berkovich and Eyal, 2015; Meyers et al., 2019; Ronen, 2020).

### The Mediating Roles of Empathic Motivation

More importantly, we discovered the mediating roles of the empathic motivation of teachers in explaining why the theories of the development of students further generate teacher empathy. Previous research indicates a close relationship between the teachers’ beliefs and their practice. This study expands this work to explore the mechanism in these psychological variables (e.g., teachers’ beliefs, teacher empathy). The relationship between teachers’ beliefs and motivation has been confirmed by numerous studies (Dweck et al., 1995; Blackwell et al., 2007; Dweck, 2012; Yeager et al., 2013). We further investigate why the theories of Students’ development are particularly predictive of the empathic motivation of teachers. Empathy is, to our knowledge, felt as a cognitive cost (e.g., the uncertainty associated with inferring information about another person’s experience), which causes people to avoid adopting empathy (Cameron et al., 2019; Ferguson et al., 2020, 2021). Therefore, empathy, an instrumental process, is tied to real-world rewards (e.g., affiliation, positive affect, etc.). This association-power motivates the adoption of empathic behavior (Zaki, 2014; Ferguson et al., 2021). When a teacher has a fixed mindset of the ability of students, they may believe that the abilities of students are fixed and unchangeable. The little reward results from empathy because no matter how hard the teacher tries to practice, the students will not change significantly. Therefore, as a primary outcome, teachers exhibit less empathy in that devoid of an obvious reward, and empathy is not significant for them (Ferguson et al., 2021).

Furthermore, previous studies indicate that there is a positive relationship between empathic motivation and empathy (Zaki, 2014; Cameron et al., 2017a, 2019; Cameron, 2018; Weisz and Zaki, 2018; Ferguson et al., 2020; Weisz et al., 2020). For example, researchers suggested that empathy, a motivational phenomenon, is a process of decision-making based on values (Cameron et al., 2019; Ferguson et al., 2020). In addition, Keysers and Gazzola (2014) suggest that empathic ability and propensity affect empathy. In other words, empathic motivation may be an important cause of empathic variations. Consistent with these previous findings and theories, this study observed a significant positive association between empathic motivation and teacher empathy, including empathic concern, perspective-taking, and fantasy for students while personal distress was not affected by empathic motivation. To our knowledge, it is generally accepted that empathy involves two information processes, namely, top-down and bottom-up. The former refers to self-regulation, while the latter refers to the automatic process (Decety and Lamm, 2006; Fan et al., 2011; Cameron et al., 2017b). Personal distress is a component of bottom-up processes, which is a self-oriented automatic aversive response to the suffering of other people (Lopez-Perez et al., 2014), and may not be influenced by cognitive control (i.e., motivation). Therefore, this is a possible explanation for the fact that the personal distress of teacher empathy was not significantly predicted by the empathic motivation of teachers and the mediation effect for the relationship between theories of Students’ development and teacher empathy-PD.

### Limitations and Implications

This study has several limitations. First, this study employed only self-report measures, which might be susceptible to response bias (e.g., social desirability). Moreover, this study was cross-sectional in design. The interpretations of the causal relationship between theories of students development, empathic motivation and empathy should be considered carefully. There is a need for future studies to examine the results using an experimental design. Second, although we try our best to recruit more Chinese teachers, the number of subjects in this study is still relatively small. Future research needs to adopt multiple approaches (for example, a combination of online and offline surveys) to expand the number of subjects. Third, some cross-cultural studies show that there are cultural differences in empathy (Zhao et al., 2019, 2021). However, we did not collect information about the cultures (i.e., teachers’ perceived professional ethics, social expectation, and educational level) that may affect teacher empathy. The relationships between cultures and teacher empathy could also be a new direction for future research. Finally, although Cronbach’s alpha coefficient of both the questionnaire of theories of Students’ development and the empathic motivation of teachers was high, and suggest a satisfactory internal consistency, we were unable to evaluate the construct validity as the number of items was too small to analyze statistically. To address these problems, a multidimensional questionnaire about the theories of the development of students and the empathic motivation of teachers needs to be developed in future studies.

Despite these limitations, the current study primarily contributed to investigating the relationship between the theories of the development of students, empathic motivation and teacher empathy and identifying an essential factor that shapes teacher empathy. This study has an important theoretical implication. Although the term “empathy” is significantly difficult to define (Assmann and Detmers, 2016; Zembylas et al., 2020), it is often viewed as an “ability” (Decety and Lamm, 2006; Shamay-Tsoory et al., 2009; Batson, 2011; Lockwood et al., 2017). Recently, however, research in the field of clinical psychology has challenged this conclusion, suggesting that individuals with mental disorders may not be impaired by their ability to empathize, but rather a lack of empathic motivation (Meffert et al., 2013; Gillespie et al., 2014). Furthermore, the theory of dissociating the ability and propensity for empathy has been proposed by Keysers and Gazzola (2014), which upholds that empathic behavior is influenced not only by the capacity for empathy but also by the motivation to empathize. Therefore, the perspective of empathy research should shift from the “ability” framework to the “ability motivation” framework. However, there is still a lack of empirical research on the relationship between empathic motivation and empathy; this study fills this research gap.

Beyond its theoretical contribution, this study also has practical implications. The empathy brain is plastic and provides a theoretical basis for the cultivation of empathy (Hein et al., 2016). Many empathic interventions focus on developing people’s ability to empathize via experience-based and expression-based interventions (Weisz and Zaki, 2017). However, empathy results from a combination of empathic capacities and motivation (Keysers and Gazzola, 2014). Therefore, studies have increasingly begun to focus on the intervention of empathic motivation via the change of mindsets (Schumann et al., 2014; Weisz, 2018), norms (Weisz et al., 2020), rewards (Ferguson et al., 2020), etc. The purpose of these interventions is to engage the empathic motivation of observers. However, the field of teacher empathy training mainly focuses on improving the ability to be empathetic (Jaber et al., 2018; Ronen, 2020). This study addresses the vital gap in the empathic motivation intervention of teachers by identifying the essential factor, which is the teachers’ beliefs about students (i.e., theories of Students’ development), that shape this psychological variable. Helping teachers improve empathic motivation through psychological interventions (e.g., changing the theories of Students’ development) should be an explicit goal for teacher education programs.

To our knowledge, this is the first study to explore the influence of the theories of Students’ development on empathic motivation and follow upon and expand the work of Schumann et al. (2014) and Weisz et al. (2020), which found that the empathy mindsets of individuals can significantly predict empathic motivation.

## Conclusion

Central to the teaching profession, teacher empathy can promote the development of students and the professional growth of teachers. Based on the motivation perspective, our finding may suggest that the teachers’ beliefs of the ability of students could predict empathic motivation and teacher empathy. Moreover, empathic motivation plays a mediating role in the theories of the development of students to expect teacher empathy, which requires more research for validation. Providing new ideas and methods to cultivate empathy for teachers, in this study, is a primary contribution.

## Data Availability Statement

The raw data supporting the conclusions of this article will be made available by the authors, without undue reservation.

## Ethics Statement

The studies involving human participants were reviewed and approved by the Zhejiang Normal University Review Board. The patients/participants provided their written informed consent to participate in this study.

## Author Contributions

YG designed the current study, collected the data, analyzed the data, and wrote this manuscript. WL proposed the research idea and demonstrate the feasibility of the method. SK participated in language polishing to ensure manuscript quality. FC and GQ joined the data analysis and the manuscript writing. All authors contributed to the article and approved the submitted version.

## Conflict of Interest

The authors declare that the research was conducted in the absence of any commercial or financial relationships that could be construed as a potential conflict of interest.

## Publisher’s Note

All claims expressed in this article are solely those of the authors and do not necessarily represent those of their affiliated organizations, or those of the publisher, the editors and the reviewers. Any product that may be evaluated in this article, or claim that may be made by its manufacturer, is not guaranteed or endorsed by the publisher.

## Footnotes

1
https://www.wjx.cn
