# Supplementary material for: The Theories of the Development of Students: A Factor to Shape Teacher Empathy From the Perspective of Motivation
Source: Front Psychol. 2021 Nov 16;12:736656. doi: 10.3389/fpsyg.2021.736656 (PMC8635053; doi:10.3389/fpsyg.2021.736656)
Supplement: Supplementary file 1 [file Data_Sheet_1.docx]

***Appendix A***

**The IRI-CT (Chinese vision)**

1.我时常作白日梦,幻想可能发生在我身上的事情。

2.对于那些没有我幸运的学生，我经常会怀有体贴、关切之情。

3.有时候我觉得很难从学生的角度看问题。

4.当有学生遇到困难时,有时我并不会很同情他们。

5.我确实会陷入小说人物的情感中。

6.在紧急状况下，我会感到担心和不安。

7.欣赏电影或戏剧时,我往往会很客观,并不会完全陷入其中。

8.在做决定前，我会去参考学生的不同意见。

9.看到有学生被利用时，我就有保护他们的想法。

10.当身处高度情绪化的情境中时，我有时会感到无助。

11.有时我会想象学生对事情的看法，从而更好地理解他们。

12.我很少会完全沉浸于一本好书或一部好电影中。

13.看到有学生受伤害时，我往往会保持平静。

14.其它学生的不幸通常不会令我不安。

15.如果我肯定自己是对的，我就不会花费许多时间去听学生的意见。

16.看完戏剧或电影后，我感觉自己好像就是其中的一个角色。

17.身处紧张的情境中，我会感到恐惧。

18.当我看到有学生受到不公平的对待时，我有时并不感到非常同情他们。

19.我经常会很有效地处理紧急事件。

20.我经常被看到的事情所深深感动。

21.我认为任何问题都有两面性，并尽量从正反两方面来考虑问题。

22.我认为自己是个非常心软的人。

23.当我观赏一部好电影时，我很容易站在某个主角的立场去感受他的心情。

24.我往往会在紧急情况下不知所措。

25.当我对一个学生生气时，我通常会试着去想一下他的立场。

26.在读有趣的故事或小说时，我想象如果故事中的事件发生在我身上，我会有什么样的感受。

27.看到有学生在紧急情况下急需帮助时，我会六神无主。

28.在批评学生之前，我会想象如果自己处于他们的立场，我会有怎样的感受。

**The IRI-CT (back-translated to English)**

1. I daydream and fantasize, with some regularity, about things that might happen to me.

2. I often have tender, concerned feelings for students less fortunate than me.

3. I sometimes find it difficult to see things from the "students'" point of view.

4. Sometimes I don't feel very sorry for students when they are having problems.

5. I really get involved with the feelings of the characters in a novel.

6. In emergency situations, I feel apprehensive and ill-at-ease.

7. I am usually objective when I watch a movie or play, and I don't often get completely caught up in it.

8. I try to look at students' sides of a disagreement before I make a decision.

9. When I see students being taken advantage of, I feel kind of protective toward them.

10. I sometimes feel helpless when I am in the middle of a very emotional situation.

11. I sometimes try to understand my students better by imagining how things look from their perspective.

12. Becoming extremely involved in a good book or movie is somewhat rare for me.

13. When I see students get hurt, I tend to remain calm.

14. Other students' misfortunes do not usually disturb me a great deal.

15. If I'm sure I'm right about something, I don't waste much time listening to students' arguments.

16. After seeing a play or movie, I have felt as though I were one of the characters.

17. Being in a tense emotional situation scares me.

18. When I see students being treated unfairly, I sometimes don't feel very much pity for them.

19. I am usually pretty effective in dealing with emergencies.

20. I am often quite touched by things that I see happen.

21. I believe that there are two sides to every question and try to look at them both.

22. I would describe myself as a pretty soft-hearted person.

23. When I watch a good movie, I can very easily put myself in the place of a leading character.

24. I tend to lose control during emergencies.

25. When I'm upset at a student, I usually try to "put myself in his shoes" for a while.

26. When I am reading an interesting story or novel, I imagine how I would feel if the events in the story were happening to me.

27. When I see a student who badly needs help in an emergency, I go to pieces.

28. Before criticizing students, I try to imagine how I would feel if I were in their place.
